# Sub-Saharan Africa's biomedical journal coverage in scholarly databases: a comparison of Web of Science, Scopus, EMBASE, MEDLINE, African Index Medicus, and African Journals Online

**DOI:** 10.5195/jmla.2023.1448

**Published:** 2023-07-10

**Authors:** Toluwase Victor Asubiaro

**Affiliations:** 1 tasubiar@uwo.ca, Alberta Law Libraries, Calgary, Alberta, Canada. Previous Affiliation: Library and Information Science Program, Faculty of Information and Media Studies, University of Western Ontario, Ontario, Canada.

**Keywords:** Biomedical research, Sub-Saharan Africa, Web of Science, Scopus, AJOL, MEDLINE, EMBASE, PubMed, African Journals Online, African Index Medicus

## Abstract

**Objective:**

This study aims to find out the coverage of biomedical journals published in Sub-Saharan Africa in four authoritative international databases-Web of Science, Scopus, MEDLINE and EMBASE and two Africa-focused scholarly databases-Africa Journals Online (AJOL) and African Index Medicus (AIM).

**Methods:**

Lists of active journals that are published in the 46 Sub-Saharan African countries were retrieved from the Ulrich periodical directory to create master journal lists. Unique journals from other databases that were not found in Ulrich were added to the master journal list. The six databases included in this study were searched for journals on the master lists.

**Results:**

Only 23 of the 46 Sub-Saharan African countries had at least one biomedical journal. Only about one-quarter (152) of the 560 biomedical journals from Sub-Saharan Africa were found in at least one of the biomedical databases. South African journals accounted for more than 50% of all the Sub-Saharan journals in the international scholarly databases. AJOL contains the highest number of biomedical journals from Sub-Saharan Africa, followed by Scopus and EMBASE. AJOL asserts its importance by covering the highest number of unique journals and having a representative number of journals in all biomedical sub-disciplines.

**Conclusion:**

The majority of studies from Sub-Saharan Africa are left out when biomedical evidence-based researchers only retrieve studies from authoritative international databases. Searching Google Scholar and the African research databases of AJOL and AIM would increase the number of studies from the region.

## INTRODUCTION

Web of Science, Scopus, MEDLINE, and EMBASE are influential research databases and sources of data for knowledge syntheses and bibliometric studies. Cochrane systematic reviews require the searching of MEDLINE and EMBASE and recommend the use of Web of Science or Scopus for citation searching [1]. One of the reasons these databases are important to knowledge syntheses and bibliometric studies is their global scope [2, 3].

However, existing studies revealed that these databases are structurally biased against research from countries outside Europe and North America, and non-English language publications [4–9]. Despite this research, there are gaps in the literature. The relevant studies on differences in geographic coverage of journals in databases concentrated on the most productive countries (such as the US, China, the UK, Germany, Japan, France, Canada, Italy, Spain, Australia, India, South Korea, Brazil, Netherlands, and Russia), omitting countries from the Middle East, Caribbean and Africa [6, 9].

The research ecosystems of some underrepresented regions in global research, especially non-English speaking regions, are well captured in their local or regional databases. Mongeon and Paul-Hus found 269 and 489 journals that are published in China from the Web of Science and Scopus respectively, the Chinese National Knowledge Infrastructure (CNKI, website address: https://oversea.cnki.net/kns?dbcode=CFLQ)contains more than 8,000 Chinese journals [6]. Mongeon and Adele also found 303 and 454 journals that are published in Japan in Scopus and Web of Science, respectively, while J-Stage (Japan's national research database, website address: https://www.jstage.jst.go.jp/journal/list/-char/en) contains more than 3,000 journals. These regional databases could supplement or provide accurate research data for the regions they cover.

### Coverage of Sub-Saharan African Biomedical Research in Scholarly Databases

Studies on the coverage of biomedical research from Sub-Saharan Africa in scholarly databases are scarce. One of the few studies that exist include Schoonbaert that focused on the coverage of biomedical research from Sub-Saharan Africa on MEDLINE database [10]. Schoonbaert found out that only 30 of 5375 journals in MEDLINE were published in Sub-Saharan Africa [10]. This is similar to Hofman et al. that found out the number of Sub-Saharan African biomedical journals indexed in MEDLINE increased from ten in 1995 to twenty-seven in 2008 [11]. Another article by Nwagwu reported that only eight of 121 local Nigerian biomedical journals were indexed in MEDLINE between 1967 and 2002 [12]. These studies are old and there are no studies that report the coverage of biomedical research from Sub-Saharan Africa in other major international and local scholarly databases. This study aims to determine the coverage of biomedical journals published in Sub-Saharan Africa in four authoritative international databases, Web of Science, Scopus, MEDLINE and EMBASE and two Africa-focused scholarly databases, Africa Journals Online (AJOL) and African Index Medicus (AIM). With this data, this study examines the extent to which scientific sources from Africa are not included in the mainstream databases and the importance of Africa-centred databases such as AIM and AJOL in capturing scientific information from Africa. Journal coverage overlap from the databases were identified to further understand the features of these databases in capturing content from Sub-Saharan Africa.

## METHODS

### Database Selection

Six databases were chosen for this study. AJOL and AIM are two databases that focused primarily on research from Africa. AJOL is an African scholarly database of journals published in Africa, with the highest collection of African journals globally. AJOL is owned and maintained as a Non-Profit Organisation in South Africa. AIM is a database of biomedical research from Africa and it is owned by the World Health Organization (WHO). MEDLINE and EMBASE are international biomedical databases. MEDLINE is maintained by the National Library of Medicine in the United States (tax-payer driven and open to all online without cost). Scopus and Web of Science are multidisciplinary scholarly databases. Many global institutional rankings and other international research assessments are based on the Web of Science and Scopus. EMBASE and Scopus are owned and maintained by Elsevier (a private corporation) in Europe, with subscription-based access. Web of Science is owned by Clarivate, a United States corporation.

### Data Collection

A master list of journals that were published in each of the Sub-Saharan African countries was created from Ulrich's Periodicals directory, AJOL, MEDLINE, Web of Science and AIM using country of publication information provided by the databases. Similar studies in the past such as Gavel and Iselidand Mongeon and Paul-Husused the Ulrich's journal list as the authoritative record of journals [6, 12]. However, it was observed during data collection that Ulrich periodical database did not capture some Sub-Saharan Africa journals that appear in AJOL, MEDLINE, Web of Science and AIM. The incomplete data on Sub-Saharan African journals in Ulrich necessitated the inclusion of the unique Sub-Saharan African journals in databases mentioned above to complement data from Ulrich. A total of 560 biomedical journals published in Sub-Saharan African countries were identified from all the databases; 509 (90.73%) journals were retrieved from Ulrich, while 52 (9.27%) other unique journals were identified from AJOL, MEDLINE, Web of Science, and AIM. South Sudan, the newest country in Sub-Saharan Africa, was not listed as a country in Ulrich, and at least one biomedical journal from South Sudan was identified in this set of 52 titles. Full details of these data collection methods are available in the supplementary materials.

### Defining Journal Country of Publication

Ulrich, AJOL, EMBASE, MEDLINE and Web of Science provided journal country of publication information. However, for journals in AIM, journal country of publication information was collected from journal websites, because AIM does not contain journal country of publication information. Some journals had more than one country of publication, and in these cases, all countries of publication were assigned to the journal using the full counting method. For instance, PAMJ was published in Kenya and Cameroun counted as one journal for Kenya and one journal for the Cameroun.

### Journal Classification

After creating master journal lists of journals published in Africa from the multidisciplinary databases (Ulrich, AJOL, and Web of Science), biomedical journals were identified using journal description and subject classification/code from the databases. The biomedical journals identified from the Ulrich, AJOL, and Web of Science were combined with those from the biomedical databases (AIM, MEDLINE, and EMBASE) and duplicates were removed. (Scopus was not considered for the creation of journal master list from Africa because it does not provide publisher's country information) The biomedical journals in the six databases were further classified into biomedical sub-disciplines manually by the author using the journal information from the databases based on classification schema from the Organization for Economic Cooperation and Development's (OECD) Frascati Manual 2015, an international standard for classifying research disciplines and sub-disciplines.

## RESULTS

There were 560 active biomedical journals from Sub-Saharan African countries. Most of the journals 98.04% (n=549/560) had English content. A total of 531 journals were monolingual; 520 journals in English language only, 7 journals in French language only, and 4 journals in Portuguese language only. Bilingual journals accounted for 5.17% (n=29/560) of all the journals; 20 titles in French and English, 7 titles in Afrikaans and English, 1 title in Arabic and English, and 1 title in Bengali and English.

### Coverage by Country

A total of 50% (n=23/46) of all the 46 Sub-Saharan African countries had at least one active biomedical journal and were included in the study. Twenty-three countries (Equatorial Guinea, Burundi, Somalia, Sao Tome and Principe, Niger, Madagascar, Guinea, Guinea-Bissau, Gambia, Benin, Cote D'Ivoire, Democratic Republic of Congo, Eritrea, Botswana, Djibouti, Gabon, Lesotho, Swaziland/Eswatini, Liberia, Reunion, Angola, Burkina Faso, and Comoros) were not included because they did not have biomedical journals. The representation of the 24 Sub-Saharan African countries with active biomedical journals on AJOL, MEDLINE, AIM, WoS, Scopus, and EMBASE is presented in Table 1. The top five countries (Nigeria, South Africa, Kenya, Ethiopia, and Ghana) out of 46 Sub-Saharan countries accounted for 90% of all the journals and had more than ten biomedical journals each.

**Table 1:** Proportion of Journals Published by the Sub-Saharan African Countries

	Country	N (%)
1	Nigeria	318 (56.79)
2	South Africa	117 (20.89)
3	Kenya	40 (7.14)
4	Ethiopia	18 (3.21)
5	Ghana	11 (1.96)
6	Uganda	8 (1.43)
7	Sudan	5 (0.89)
8	Mozambique	4 (0.71)
9	Tanzania	6 (1.07)
10	Zambia	5 (0.89)
11	Cameroun	8 (1.43)
12	Malawi	3 (0.54)
13	Senegal	3 (0.54)
14	Zimbabwe	3 (0.54)
15	Republic of the Congo	2 (0.36)
16	Namibia	2 (0.36)
17	Mali	1 (0.18)
18	Mauritius	1 (0.18)
19	Rwanda	1 (0.18)
20	Seychelles	1 (0.18)
21	Sierra Leone	1 (0.18)
22	Togo	1 (0.18)
23	South Sudan	1 (0.18)

Only 152 (27.14%) of these 560 active journals were included in at least one of the six databases included in this study. AJOL (13.93%, n=78/560) and Scopus (10.36%, n=58/560), followed by EMBASE (9.11%, n=51/560) and AIM (9.11%, n=51/560) contained the highest number of journals from the Sub-Saharan African countries. Nigeria had almost three times more biomedical journals than South Africa, but South Africa had more biomedical journals that were indexed in all six databases included in this study. For instance, the number of South African journals in Scopus, WoS, and MEDLINE was more than the number of journals from all other Sub-Saharan African countries put together. In addition, 50% of all the journals from Sub-Saharan African countries that were indexed in EMBASE are from South Africa. Journals from 12 of the 46 (21.42%) Sub-Saharan African countries are included in AIM and AJOL, followed by ten (17.86%) in EMBASE, nine (16.07%) in Scopus, seven (12.5%) in each of WoS and MEDLINE.

### Coverage by Sub-discipline

In the 560 journals, the subdisciplines were covered as follows: Clinical Medicine (49.82%, n=279), followed by Basic Medicine (39.82%, n=223), Health Sciences (37.86%, n=212), Biological Sciences (27.32%, n=153), other Medical Sciences (29.11%, n=163), Veterinary (4.64%, n=26) and Medical Biotechnology (31.61%, n=177). Detailed information about sub-discipline coverage is available in the Supplementary materials.

### Overlap Between the Databases

Of the 152 unique journals found in the six databases, further analysis was performed to find out the number of journals that were unique (found in one, not in any other) to each of the six databases. AJOL had the highest number of unique journals, with 50 (32.90%) of its journals not found in any of the other databases, followed by AIM with 16 (10.53%) unique journals. Other databases have fewer unique journals: EMBASE (5, 3.29%), Web of Science (3, 1.97%), Scopus (2, 1.32%), and MEDLINE (1, 0.66%).

**Table 2:** Percentage of all the Journals from a Sub-Saharan African Country in the selected Databases

	Country	AJOL	MEDLINE	AIM	WoS	Scopus	EMBASE
		N (%)	N (%)	N (%)	N (%)	N (%)	N (%)
1	Nigeria	40 (12.58)	2 (0.63)	15 (4.72)	3 (0.94)	8 (2.52)	11 (3.46)
2	South Africa	13 (11.11)	12 (10.26)	19 (16.24)	39 (33.33)	39 (33.33)	30 (25.64)
3	Kenya	8 (20.00)	2 (5.00)	3 (7.50)	3 (7.50)	3 (7.50)	2 (5.00)
4	Ethiopia	5 (22.73)	1 (4.55)	3 (13.64)	2 (9.09)	3 (13.64)	1 (4.55)
5	Ghana	2 (18.18)	2 (18.18)	1 (9.09)	1 (9.09)	1 (9.09)	2 (18.18)
6	Uganda	1 (12.50)	2 (25.00)	1 (12.50)	2 (25.00)	1 (12.50)	2 (25.00)
7	Sudan	0	0	0	0	0	0
8	Mozambique	0	0	0	0	0	0
9	Tanzania	2 (33.33)	0	0	0	1 (16.67)	1 (16.67)
10	Zambia	1 (20.00)	0	0	0	0	0
11	Cameroun	4 (50.00)	0	4 (50.00)	0	0	0
12	Malawi	1 ()	1 (33.33)	1 (33.33)	1 (33.33)	1 (33.33)	1 (33.33)
13	Senegal	0	0	0	0	0	0
14	Zimbabwe	0	0	0	0	0	0
15	Republic of the Congo	0	0	1 (50.00)	0	0	0
16	Namibia	0	0	0	0	0	0
17	Mali	0	0	1 (100.00)	0	0	1 (100.00)
18	Mauritius	0	0	0	0	0	1 (100.00)
19	Rwanda	0	0	1 (100.00)	0	1 (100.00)	0
20	Seychelles	0	0	0	0	0	0
21	Sierra Leone	0	0	0	0	0	0
22	Togo	0	0	0	0	0	0
23	South Sudan	1	0	1 (100.00)	0	0	0
	Total	78 (13.90)	23 (4.11)	51	51 (9.03)	58 (10.36)	52 (9.2)

Since AJOL and AIM had the highest number of unique journals, these two databases were used as reference databases to compare overlap with the other four databases. The result in Table 3 shows that the overlap between AJOL and the other databases with an international scope is below 18% (minimum= 9.78%, maximum= 17.58%), compared to AIM with higher overlap ratios with the international databases above 22% (minimum= 23.73%, maximum=25.93%). Results of the comparison between the four international databases-WoS, Scopus, MEDLINE and EMBASE (using Scopus and the reference index) is presented on Table 4. The overlap between the three (Web of Science, Scopus and EMBASE) of the four databases with international scope is high, with MEDLINE having the smallest overlap and least number of unique journals. This suggests WoS, Scopus, and EMBASE are not significantly different from each other. Though overlap between MEDLINE and the other international databases is small, it offers few number of unique journals as compared with other databases.

**Table 3:** Comparison of coverage overlap of the 152 Sub-Saharan African journals indexed in Africa-focused Academic Databases and Global Citation Database

Reference Database (N titles indexed)	Query Database (N titles indexed)	Overlap in Titles
AJOL (78)	MEDLINE (23)	9.78%
AJOL (78)	WoS (51)	15.04%
AJOL (78)	Scopus (58)	17.54%
AJOL (78)	Embase (52)	11.02%
AJOL (78)	AIM (51)	13.04%
AIM (51)	MEDLINE (23)	23.73%
AIM (51)	WoS (51)	25.93%
AIM (51)	Scopus (58)	24.71%
AIM (51)	Embase (52)	22.62%

**Table 4:** Comparison of coverage overlap of the 152 Sub-Saharan African journals indexed in the global citation databases

Reference Database (N titles indexed)	Query Database (N titles indexed)	Overlap in Titles
Scopus (58)	WoS (51)	60.61%
Scopus (58)	Embase (52)	52.86%
Scopus (58)	MEDLINE (23)	28.33%

## DISCUSSION

### Summary of Findings

This study focused on finding out the coverage of biomedical journals that are published in the 46 Sub-Saharan African countries by six scholarly databases (two multidisciplinary and international databases WoS, Scopus, two international biomedical databases MEDLINE, EMBASE, and two Africa-focused databases AIM and AJOL). Only 27.14% (152/560) of the biomedical journals were included in at least one of the sampled databases. No single database covered more than 14% (N=78) of the biomedical journals. Only 152 (27.14%) of these 560 active journals were included in at least one of the six databases included in this study. AJOL (13.93%, n=78/560) and Scopus (10.36%, n=58/560), followed by EMBASE (9.11%, n=51/560) and AIM (9.11%, n=51/560) contained the highest number of journals from the Sub-Saharan African countries. WoS, Scopus, EMBASE, and MEDLINE contain 96 (17.14%) biomedical journals from Sub-Saharan Africa. There are 56 (11.2%) journals in AJOL and AIM that were not indexed in WoS, Scopus, EMBASE, or MEDLINE.

### Practical Implications

1. Comprehensive Search of Sub-Saharan African Biomedical Journals

Searching any one of these databases would retrieve only a fraction of studies from Sub-Saharan Africa. This is against the ethos of biomedical evidence-based research where every valuable piece of evidence or study is important for a robust, non-biased and reliable knowledge synthesis. This study shows the importance of considering the African research databases of AJOL and AIM when conducting comprehensive searches. Searching the four international citation databases? WoS, Scopus, EMBASE and MEDLINE would retrieve 96 biomedical journals from Sub-Saharan Africa while missing the 56 journals (58.33%) that are only found in AJOL and AIM Google Scholar was not included in this study, but it is another source that could potentially index more journals from Sub-Saharan Africa.

The search capabilities of AJOL and AIM create a barrier to comprehensive searching of these databases. AJOL uses a simple Google search within the journal contents, while AIM's search interface does not accept complex queries like those that include adjacency searching. However, considering the important content in these databases, it is worth the effort of including the African-focused databases in a comprehensive search. In the long run, there is a need for Africa to develop a citation database that could capture scientific knowledge in AJOL, AIM, and other local Africa-focused sources. Like WoS and Scopus, the development of the proposed citation database for Africa could involve the constitution of journal selection board members that would apply inclusion criteria for selecting journals into the database. This step is necessary to ensure the credibility of the content of the databases.

2. Paid Vs Open Access Citation Databases and the Role of the World Health Organization

There is a conundrum in choosing between the paid and open access citation databases for access to local Sub-Saharan African biomedical journals by librarians and stakeholders in Sub-Saharan Africa. EMBASE (subscription-based-online access) contains nearly three times the number of Sub-Saharan African journals in MEDLINE (free online access). AJOL (with a basic search interface) also covers about 50% more journals than AIM (with a more advanced search interface). The databases that provide free online access to biomedical journals from Sub-Saharan Africa are either narrow (i.e. AIM or MEDLINE through PubMed) or little broader but without advanced search interfaces (i.e. AJOL). Those with subscription-based access provide robust search interfaces but cover a fraction of the journals (EMBASE, WoS, and Scopus). There is a need for the World Health Organization (funder of AIM) to re-evaluate its curation strategy into AIM to cover journals in AJOL and other local African biomedical journals. Building a biomedical database that covers a wider range of journal content would create a place for librarians and other stakeholders to search regardless of their access to subscription databases like WoS, Scopus, and EMBASE. The World Health Organization) rely on international databases as sources of scientific information. This implies that the stakeholders have access to only a fraction of biomedical research from the region for decision-making, missing out on many potentially beneficial biomedical studies that are published in local Sub-Saharan journals and are not indexed by international databases.

3. Role of South Africa and Nigeria

Nigeria and South Africa outstandingly contributed to publishing biomedical journals in Sub-Saharan Africa, more than all other countries in the region put together. The role of South Africa and Nigeria as the foremost in scientific productivity in the African continent is well documented in the literature [1–5]. South Africa and Nigeria are the biggest economies in Sub-Saharan Africa, and this study reveals two contrasting trajectories of these two countries in research and publishing. Nigeria publishes more than half of all biomedical journals from Sub-Saharan Africa, whereas South Africa publishes the highest number of journals that were indexed in international scholarly databases, more than all other Sub-Saharan African countries put together. The representation of journals from South Africa in international databases could be as a result of its relatively rich investment in Africa. South Africa dominates research in Sub-Saharan Africa and its universities are the highest ranked in the region [14–16], Nigeria's population is more than three times bigger than that of South Africa, but South Africa spends six times more on higher education [17]. Researchers in South Africa also enjoy more conducive environment for research (stable electricity supply, research infrastructure and social amenities) and stability than those in Nigeria where there face insecurity and economic instabilities. While universities in South Africa have uninterrupted academic sessions, closure of universities in Nigeria is a usual occurrence, with the latest closure spanning ten months in 2022.

## LIMITATIONS OF THE STUDY

One of the limitations of this study is the inclusion of only six databases. There are other international databases such as Open Alex, Dimensions and CrossRef where journals that were not covered in the sampled databases could be indexed. Some steps like searching for journals were manually executed; manual processes are prone to human error.

The following are suggested as future studies to provide evidence-based information on why biomedical journals from Africa is under-represented in recognized international citation databases:

- It is recommended that journal editors from various parts of the world be surveyed about how fair they believe international research indexing databases to be when selecting academic journals for publication worldwide. More understanding might result from a comparison of the editors' perspectives from various regions.
- Future research should examine how the worldwide academic databases' selection criteria may adversely affect the likelihood that journals from Sub-Saharan Africa will be selected for inclusion in international research indexing databases.
- Future research is recommended to determine how neo-colonialism affects international academic publishing and indexing.

This study shows that the majority of biomedical journals that are published in Sub-Saharan Africa are not indexed in the conventional research indexing databases-Web of Science, Scopus, EMBASE and PubMed. Searching any one of these databases would retrieve approximately 10% (or less) of the biomedical journals that are published in the region. The importance of considering local academic databases like AJOL and AIM was highlighted, with AJOL and AIM containing the highest number of unique journals that were not found in any of Web of Science, Scopus, PubMed and EMBASE. If Web of Science, Scopus, EMBASE and PubMed are all searched, in addition to searching the two local databases-AJOL and AIM, there is a chance of retrieving about 27% of the biomedical journals from the region, about thrice the amount that could be retrieved from any one of the four international and conventional research databases. Therefore, it is recommended that librarians incorporate AJOL and AIM into their comprehensive searches on subjects pertaining to Sub-Saharan Africa. Further research needs to be conducted to investigate how to increase the visibility of Sub-Saharan African biomedical journals.

## Data Availability

Data is freely available on Mendeley Data with doi: 10.17632/52pncd8zmy.2.
