# Supplementary material for: Sub-Saharan Africa's biomedical journal coverage in scholarly databases: a comparison of Web of Science, Scopus, EMBASE, MEDLINE, African Index Medicus, and African Journals Online
Source: J Med Libr Assoc. 2023 Jul 10;111(3):696–702. doi: 10.5195/jmla.2023.1448 (PMC10361549; doi:10.5195/jmla.2023.1448)
Supplement: Supplementary file 1 — Appendix A [file jmla-111-3-696-s01.pdf]

Appendix A

Methodology

Journal lists of all the 46 Sub-Saharan African countries were retrieved manually from Ulrich using the "country of publication" field in the advanced search interface. Delimiters were used to limit the retrieved results from Ulrich to periodicals in the journal categories and with active status. Ulrich contains multiple records of journals with different formats (eg. online and print), or languages, resulting in duplications. Duplicates were removed from the retrieved results.

A master journal list for Web of Science was created from four of its indexes comprising of the Science Citation Index Expanded (SCIE), the Social Science Citation Index (SSCI) and the Arts and Humanities Citation Index (A&HCI) and Emerging Sources Citation Index (ESCI). Master journal lists for Scopus, EMBASE and MEDLINE databases were downloaded from their respective websites. A master journal lists for AJOL was not available on the publishers' website as a downloadable file, though journals were organized by country of publication. Therefore, the master journal list for AJOL was created manually by extracting journal information from the publishers' websites (https://www.ajol.info/index.php/ajol). Only active journals were included in the study, where active journals were defined as journals that have published at least an issue in 2021 or 2020. The master journal list for AIM was not available as well. A journal master list was created for AIM by downloading the whole database of AIM with the source (journal names). The journals were sorted to identify unique journal names, , .and journals that were not indexed in the AIM database in 2020 or 2021 removed. This study was not considered for ethics review because data used was collected from publicly available records. Journal lists from Ulrich, Web of Science, AJOL, AIM, EMBASE, MEDLINE and Scopus were collected between in April and May 2021.

Conflicting journal country of publication information was noticed in the case of Pan-African Medical Journal (PAMJ). MEDLINE and Web of Science listed Uganda as the journal country of publication while the website of the journal listed Kenya and Cameroun as the countries of publication. The journal country of publication information on the journal website was considered for the analysis.

The Sub-disciplines were majorly drawn from the Medical and health sciences class- Basic Medicine (subject areas: Medical Biochemistry, Anatomy, Pathology, Immunology, Hematology,

Parasitology, Physiology, Laboratory Medicine, Pharmacy, Pharmacology and Virology), Clinical Medicine (subject areas: Anesthesia, Urology, Nephrology, Paediatrics, dentistry, Obstetrics and Gynaecology, Surgery, Oncology, Radiology, Psychiatry, Physiotherapy, Ophthalmology, Oto-Rhino-Laryngology, Occupational Therapy, Nursing and Orthopaedics), Health Sciences (subject areas: Public Health, Nursing, Epidemiology, Health Policy and Management, Human Nutrition, Health Promotion and Education), Medical biotechnology (subject areas: Biotechnology, Bioengineering, Computational Biomedicine, Medical Laboratory Technology), and Other medical science (subject areas: Medical Law, Forensic Science, Medical Laboratory Science, Alternative medicine). Other sub-disciplines were Biological sciences (subject areas: Botany, Zoology, Ornithology) from the Natural sciences discipline and veterinary sciences from the Agricultural and veterinary sciences discipline.

Result

A comparison between Nigeria and South Africa was included to understand the country coverages of these databases at the sub-discipline level using visualization in Figure 1. Other countries were left out because data was not large enough for this category of comparison. Nigeria had more biomedical journals in total, about three times more than South Africa. Clinical medicine dominated South African biomedical journals in five of the six databases- MEDLINE, Web of Science, Scopus, AIM and EMBASE, except in AJOL. While South African journals are specialized, concentrating majorly in Clinical Science, followed by Health Sciences and Biological Sciences. In contrast, biomedical journals from Nigeria are multidisciplinary (eg in Web of Science and AIM) where there are no significant differences between the biomedical sub-disciplines. Overall, Clinical Sciences, Basic Medical Sciences and Health Sciences are the sub-disciplines with the highest number of journals in Nigeria.

Figure 1: Distribution of Biomedical Journals published in all the Sub-Saharan African countries, Nigeria and South Africa by Sub-Disciplines

Figure 1a: Journals published in all the Sub-Saharan African countries

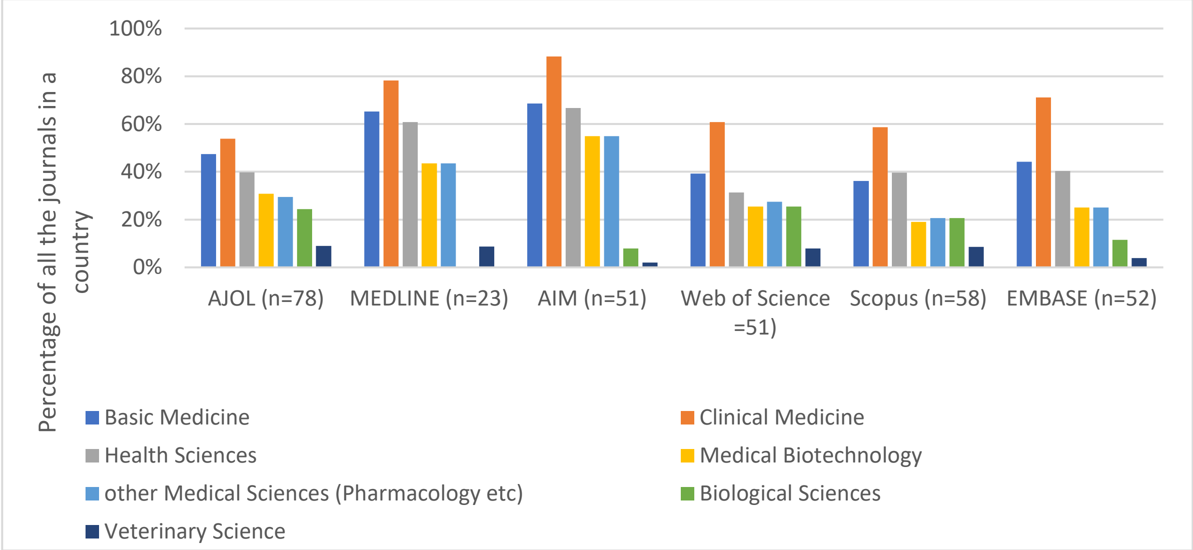

Figure 1b: Journals published in Nigeria

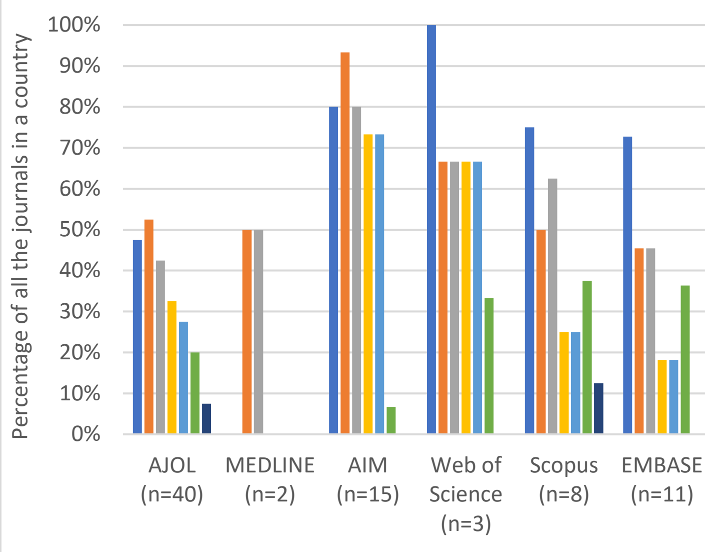

Figure 1c: Journals published in South Africa

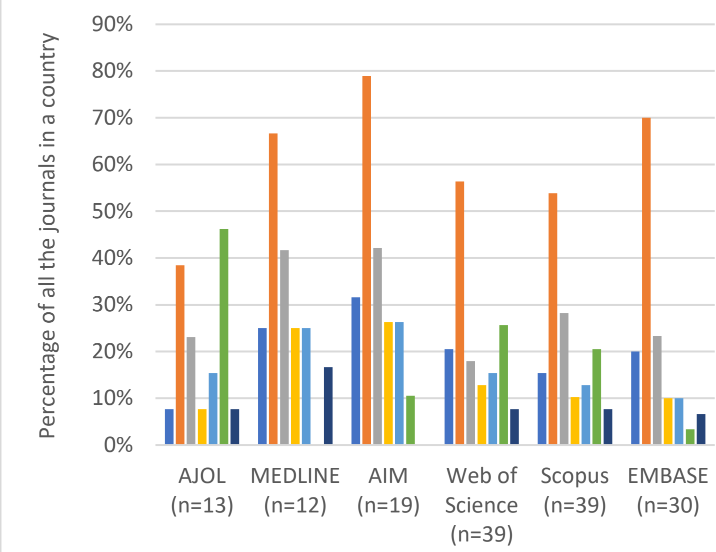

Basic Medicine Clinical Medicine Health Sciences Medical Biotechnology

Other Medical Sciences Biological Sciences Veterinary Sciences

Table 3: Journal Distribution by Country and Sub-Discipline

S/N	Country	Biomedical	Basic Medicine	Clinical Medicine	Health Sciences	Medical Biotechnology	other Medical Sciences	Biological Sciences	Veterinary Science
1	Nigeria	318	136	133	104	21	37	77	13
2	South Africa	117	17	69	29	3	10	21	3
3	Kenya	40	19	24	18	1	6	7	1
4	Ethiopia	22	6	9	6	0	4	2	4
5	Ghana	11	4	8	6	2	2	1	0
6	Uganda	8	2	2	3	0	3	2	0
7	Sudan	5	2	4	3	0	0	0	0
8	Mozambique	4	1	2	2	0	0	0	1
9	Tanzania	6	3	3	4	0	0	0	1
10	Zambia	5	0	2	2	0	0	0	0
11	Cameroun	8	6	6	1	0	1	2	0
12	Malawi	3	2	1	1	0	0	1	0
13	Senegal	3	2	3	2	0	0	0	0
14	Zimbabwe	3	1	1	1	0	1	0	1
15	Republic of the Congo	2	2	2	2	0	0	0	0
16	Namibia	2	0	0	0	0	0	2	0
18	Mali	1	1	1	1	0	0	0	0
19	Mauritius	1	1	1	1	0	0	0	0
20	Rwanda	1	1	1	1	0	0	0	0
21	Seychelles	1	0	0	0	0	0	1	0
22	Sierra Leone	1	0	0	0	0	0	1	0
23	Togo	1	0	0	0	0	0	1	0
24	South Sudan	1	1	1	0	0	0	0	0
	Total	208	274	188	27	64	118	24	208

Figure 1b and Figure 1c show a contrast between biomedical journals that were published in South Africa and Nigeria. AJOL is the database with highest number of Nigerian journals while other databases housed South African journals than AJOL and MEDLINE. Web of Science and Scopus indexed more South African journals than other databases, unlike Nigeria. South African biomedical journals were better represented in other databases than AJOL. Figure 1a, Figure 1b and Figure 1c show the few Veterinary Sciences journals (4.29%) are the least visible journals in all the databases.
